# Comparative Analysis of Salivary and Serum Inflammatory Mediator Profiles in Patients With Rheumatoid Arthritis and Periodontitis

**DOI:** 10.1155/mi/7739833

**Published:** 2025-03-20

**Authors:** Carina Fei, Kaja Eriksson, Guozhong Fei, Luis Fernando Delgado Zambrano, Shigufta Syed, Caroline Lindström, Martina Ericson, Mehrad Mohammadi, Georgios Tsilingaridis, Amel Guenifi, Rikard Holmdahl, Leif Jansson, Tülay Yucel-Lindberg

**Affiliations:** ^1^Department of Dental Medicine, Division of Pediatric Dentistry, Karolinska Institutet, Huddinge, Sweden; ^2^Center for Rheumatology, Academic Specialist Centre, Stockholm, Sweden; ^3^Rheumatology Unit, Karolinska University Hospital, Huddinge, Sweden; ^4^Department of Gene Technology, School of Engineering Sciences in Chemistry, Biotechnology and Health, KTH Royal Institute of Technology, Science for Life Laboratory, Stockholm, Sweden; ^5^Department of Dental Medicine, Division of Periodontology, Karolinska Institutet, Huddinge, Sweden; ^6^Center of Pediatric Oral Health Research, Stockholm, Sweden; ^7^Department of Medical Biochemistry and Biophysics, Division of Medical Inflammation Research, Karolinska Institutet, Stockholm, Sweden; ^8^Folktandvården Eastmaninstitutet, Stockholm, Sweden

**Keywords:** inflammatory mediators, periodontitis, rheumatoid arthritis, saliva, serum

## Abstract

**Background:** Periodontitis (PD) and rheumatoid arthritis (RA) are chronic inflammatory conditions, characterized by dysregulated immune response and excessive production of inflammatory mediators. The oral disease PD is triggered by periodontal pathogens, leading to the destruction of tissues surrounding the teeth, whereas RA is a systemic autoimmune disease primarily affecting the joints. The objective of this study was to investigate the prevalence of PD and map the profile of salivary and serum inflammatory mediators of patients with RA, with respect to periodontal severity (PD stage II and PD stage III/IV).

**Methods:** For this cross-sectional cohort study, 62 patients diagnosed with RA were recruited. All participants underwent a full-mouth dental examination. Levels of various inflammatory mediators, including tumor necrosis factor (TNF) superfamily proteins, interferon (IFN) family proteins, regulatory T cell (Treg) cytokines, and matrix metalloproteinases were determined in saliva and serum samples from each participant using a human inflammation multiplex immunoassay panel.

**Results:** In the current RA cohort, all participants were diagnosed with PD, of which 35.5% were classified as PD stage II and 64.5% as PD stages III/IV. Inflammatory mediator levels were significantly higher in both saliva and serum samples from patients with RA and PD stages III/IV, compared to those with RA and stage II within the same cohort. These included higher serum levels of sCD30, IL-10, IL-19, osteopontin and elevated salivary levels of BAFF/TNFSF13B and IFN-*α*2. Additionally, APRIL/TNFSF13 levels were increased in both saliva and serum.

**Conclusions:** Among the studied patients with RA, the majority exhibited severe PD (stage III/IV), underscoring the importance of periodontal prophylaxis and treatment for this group of patients. Higher levels of inflammatory mediators were observed in both saliva and serum in those with PD stages III/IV, suggesting a potential link between the severity of PD and systemic inflammation in RA. Further research is needed to explore the clinical implications of these findings.

## 1. Introduction

Periodontitis (PD) is a chronic inflammatory disease that affects the structures surrounding and anchoring the teeth to the mandibular and maxillary bone. With an estimated prevalence of 42%–72.4% in the adult population, PD is one of the most prevalent chronic inflammatory diseases [1, 2]. Pathogenesis of PD is associated with a profound shift in the composition of subgingival bacterial communities and the accumulation of periodontal pathogens in the tooth-supporting tissues [3, 4]. It is known that periodontal pathogens collectively initiate an inflammatory response leading to the production of numerous inflammatory mediators, cytokines, proteolytic enzymes, prostaglandins, and toll-like receptors which can cause the destruction of alveolar bone and connective tissue [5, 6]. Proinflammatory cytokines associated with PD include interleukin (IL)-1, IL-6, IL-12, IL-17, IL-18, IL-21, tumor necrosis factor (TNF)-*α*, and interferon (IFN)-*γ* [5, 7]. Regulatory cytokines such as IL-4, IL-1Ra, and IL-10 are also suggested to be involved in the pathogenesis of PD [8]. Moreover, PD has previously been recognized for its systemic implications, suggesting a relationship to several systemic diseases such as cardiovascular diseases, diabetes mellitus, and rheumatoid arthritis (RA) [9–11].

RA is a chronic inflammatory disease with a prevalence of 1% of the world population, predominantly affecting women [12]. This disease is characterized by synovial joint inflammation and irreversible destruction of cartilage and underlying bone in the joints [13]. Synovial joint inflammation is associated with the accumulation of inflammatory mediators, including TNF, IL-1, IL-6, IL-16, and IL-17, driving the systemic inflammation [14]. Autoantibodies known as anticitrullinated protein antibodies (ACPAs) are established biomarkers of RA, present in ~70% of patients, often years before the clinical onset of the disease [15]. These autoantibodies target the citrulline sidechain, a posttranslational modification where arginine is converted to citrulline, exposed on proteins [15]. The oral pathogen *Porphyromonas gingivalis*, associated with PD, is the only identified bacteria known to catalyze citrullination, potentially contributing to the generation of ACPAs [16, 17]. Specifically, *P. gingivalis* infection might contribute to the development or exacerbation of RA by inducing the production of citrullinated proteins, which can trigger the autoimmune response in genetically susceptible individuals [18].

Multiple studies have suggested an epidemiological association between PD and RA [19, 20]. Both diseases share a similar profile of cytokines and inflammatory mediators that are present in synovial joints of patients with RA as well as in periodontal tissue in individuals with PD [17]. In addition, studies have reported an increased risk of PD in patients diagnosed with RA and vice versa an increased incidence of RA in patients with PD [21–23]. Previous research has shown a high prevalence of severe PD in patients with RA ranging from 29% to 48% [24, 25], compared to an estimated 7.8%–17.6% in general population [1, 2]. However, these observations were based on previous periodontal classifications. In this study, we aimed to assess the prevalence of PD in patients with RA, according to the currently used periodontal classification scheme, which encompasses both the severity and extent of the disease through stages [26]. Additionally, we aimed to profile salivary and serum inflammatory mediators in patients with RA, in relation to periodontal disease, according to the current classification stages.

## 2. Patients and Methods

### 2.1. Study Population and Patients

For this cross-sectional cohort study, 78 patients with RA were recruited. The inclusion criteria were a confirmed RA diagnosis and an age of 18 years or older. Exclusion criteria included recent antibiotic use or periodontal treatment within the past 3 months, pregnancy, lactation, or unwillingness to participate. As a result, 16 participants were excluded from the study. All participants were recruited from the Rheumatology Department at Karolinska University Hospital, Huddinge. Data regarding ACPA, rheumatoid factor (RF), C-reactive protein (CRP), and disease activity score (DAS-28) were collected from the responsible rheumatologist. In addition, all participants answered questionnaires regarding general health, medications, tobacco-smoking habits, personal information, e.g., level of education, and a health assessment questionnaire (HAQ). A dentist (K.E), calibrated by a periodontist (L.J), conducted a full dental examination of all participants at the Department of Dental Medicine at Karolinska Institutet, Huddinge. Dental examination included clinical periodontal variables at six sites on each tooth measuring: probing pocket depth (PPD) ≥4 mm, bleeding on probing (BOP), and clinical attachment loss (CAL). Plaque index (PI) was recorded at four sites on each tooth, excluding third molars. In addition, missing teeth, furcation involvement, and mobility were recorded. Dental radiographic examination was performed which comprised of bitewing X-rays and one orthopantogram. Periodontal diagnosis was based on the current periodontal classification scheme according to AAP/EFP [26]. Sample size calculation was performed using a statistical power analysis tool, based on data from our previous study [27], including inflammatory mediator levels in saliva and serum from patients with RA and PD (severe/moderate PD and gingivitis/mild PD). The analysis estimated a minimum of 59 participants to achieve a significance level of 0.05 and a statistical power of 0.95 (*a* = 0.05, *β* = 0.95).

### 2.2. Collection of Saliva and Serum Samples

Stimulated saliva and serum samples were collected from each study participants. The participants were not allowed to drink or eat 1 h prior to the examination, and saliva was collected before initiation of the oral examination. Stimulated saliva was collected by chewing on paraffin wax (1 g, Ivoclar Vivadent Liechtenstein) for a duration of 2 minutes, as previously described [28, 29]. After measuring the volume and flow rate, it was placed into 15 ml falcon tubes and centrifuged at 5°C with 500 × *g* for 10 min. Thereafter, the supernatants were aliquoted to 1.5 ml tubes and stored at −80°C until analysis of inflammatory mediators. Salivary total protein concentrations were measured using the DC (detergent compatible) protein assay (Bio-Rad Laboratories AB) following the manufacturer's instructions. Blood samples were collected in BD Vacutainer SST tubes (MediCarrier AB, Stockholm, Sweden), which were then left at room temperature for 30 min to allow clot formation. The serum samples were then centrifuged at 200× g for 10 min at 20°C and aliquoted in 1.5 ml tubes at −80°C until further processing and analysis.

### 2.3. Analysis of Inflammatory Mediators in Saliva and Serum Samples

Levels of inflammatory mediators in saliva and serum samples were determined using human inflammation multiplex Immunoassay panel (Bio-Rad Laboratories, Hercules, CA, USA), which includes TNF superfamily proteins, IFN family proteins, regulatory T cell (Treg) cytokines, and matrix metalloproteinases (MMPs). The samples falling below the sensitivity level of the assay were replaced with the limit of detection (LOD) specific to each respective analyte, as specified by the manufacturer (Bio-Rad Laboratories). The analyzed inflammatory mediators (detection limits in pg/ml) were APRIL/TNFSF13 (190), BAFF/TNFSF13B (34.7), sCD30/TNFRSF8 (1.0), sCD163 (16.8), Chitinase 3-like 1 (10.3), gp130/sIL-6R*β* (16.9), IFN-*α*2 (0.7), sIL-6R*α* (1.5), IL-8 (2.7), IL-10 (0.6), IL-11 (0.05), IL-12/p70 (0.1), IL-19 (0.2), IL-20 (3.6), IL-22 (1.1), IL-26 (1.2), IL-27/p28 (0.1), IL-29/IFN-*λ*1 (1.6), IL-32 (12.3), IL-34 (51.9), IL-35 (3.7), LIGHT/TNFSF14 (10.2), MMP-1 (33.7), MMP-2 (39.7), MMP-3 (28.5), osteocalcin (23.4), osteopontin (91.3), Pentraxin-3 (0.8), sTNF-R1 (0.2), sTNF-R2 (3.2), TSLP (0.8), and TWEAK/TNFSF12 (0.5), as previously described by Eriksson et al. [27]. The following inflammatory mediators were excluded from further analysis due to the majority of samples being below the LOD: in serum (IFN-*α*2, IL-8, IL-11, IL-12, IL-20, IL-22, IL-27, IL-32, IL-34, LIGHT/TNFSF14, MMP-1, and TSLP) and in saliva (sCD30/TNFRSF8, IL-11, IL-12, IL-20, IL-27, IL-29/IFN-*λ*1, IL-32, LIGHT/TNFSF14, MMP-1, MMP-2, osteocalcin, osteopontin, and TSLP).

### 2.4. Statistical Analysis

Descriptive statistics and statistical analyses were performed using R.4.2.0. and SPSS (IBM SPSS Statistics 21.0; SPSS Inc). For comparison between the groups, Wilcoxon rank sum test was used for numerical variables, and Chi-square test was used for categorical variables. The Spearman correlation analysis was adopted to investigate the correlations between RA duration and the demographic/anamnestic variables. For the logistic regression model, backward procedures were used with the occurrence of bleeding pockets (sites with PPD ≥4 mm and BOP ≤3.0, >3.0), bleeding index (BI) (≤30, >30), and PD stage III/IV versus stage II as the dependent variables and inflammatory mediators in saliva/serum and potential confounders as independent variables, included in the model if *p* < 0.10. Potential confounders were defined as variables correlated (Spearman correlation *p* < 0.010) to at least one of the inflammatory mediators and the dependent variable. The results were adjusted for age, ACPA status, and smoking status (current/former smokers). Inflammatory mediator levels were log-transformed to achieve normality. The level of statistical significance was set at *p* < 0.05. Random Forest analysis was done using R package randomForest v4.7.1.1. The randomForest mtry parameter (i.e., number of variables randomly sampled as candidates at each split) was tune with the function tuneRF, and the MeanDecreaseGini values were used as indicative of the feature importance. The train/test data split for Random Forest model calibration was carried out using a stratified sampling with the function initial split of the R package tidymodels v.1.1.1. To evaluate the model prediction power, two approaches were used: test dataset validation and when using the training dataset, an out-of-bag (OOB) approach [30]. Receiver operating characteristic (ROC) and area under the curve (AUC) were calculated using the R package pROC [31]. Principal coordinate analysis (PCoA) was performed using the R package vegan v2.6-4. The analysis was conducted using all periodontal variables and inflammatory mediators in saliva and serum samples. Before PCoA, the data were scaled by dividing each column by its standard deviation while retaining the original mean. To visualize correlations between salivary and serum inflammatory mediators, sparse partial least squares discriminant analysis (sPLS-DA) method was conducted, using mixOmics R package. Prior to the sPLS-DA analysis, the salivary and serum inflammatory mediator data was normalized to zero mean and unit variance.

## 3. Results

### 3.1. Characteristics of the Study Population

A total of 62 participants diagnosed with RA were included in this study. Demographic data collected from all participants, incorporating both the clinical examination and the health questionnaire, are shown in Table 1. Enrolled participants were predominantly women (90%), with a median age of 64 years and a median RA duration of 8.5 years from diagnosis of RA. All study participants exhibited the presence of PD; 35.5% (*n* = 22) were diagnosed with stage II, 48.4% (*n* = 30) with stage III, and 16.1% (*n* = 10) with stage IV. Among the participants, 8.1% (*n* = 5) were diagnosed with PD grade A, 80.1% (*n* = 50) with PD grade B, and 11.3% (*n* = 7) were diagnosed with PD grade C. As for comorbidities, 40.3% were diagnosed with temporomandibular joint (TMJ) disorders, 21% with gastrointestinal disorders, 20.1% with asthma, 19.4% with high blood pressure, and 12.9% with cardiac disorders. The majority of the study participants were positive for ACPA (77.4%) and RF (64.5%).

When comparing PD stages II with III/IV, demographic differences were observed. The median age in the group with stage III/IV was higher as compared to the group with stage II (Table 1). Concerning smoking, more former/current smokers were present in the group with the severe forms of PD (stage III/IV:77.5% *vs* stage II:45.5%, *p*=0.01). Furthermore, the prevalence of TMJ also differed between the groups (stage II:59.1% *vs* stage III/IV:30%, *p*=0.049). There were no significant differences between the two groups regarding gender, body mass index (BMI), RA duration, alcohol consumption habits, or level of education. Additionally, RA duration was not correlated with these variables. Furthermore, no significant differences were observed between the two groups regarding the use of analgesics, nonsteroidal anti-inflammatory drugs (NSAIDs), disease-modifying antirheumatic drugs (DMARDs), biological disease-modifying antirheumatic drugs (bDMARDs), or glucocorticoids. Similarly, comorbidities such as diabetes, cardiac and vascular disorders, gastrointestinal disorders, osteoporosis, asthma, Sjögren's syndrome, and liver diseases did not differ significantly between groups. Moreover, no significant differences were found in ACPA, RF, DAS-28, HAQ, or CRP when comparing RA patients with PD stage II to those with PD stage III/IV (Table 1).

### 3.2. Clinical Periodontal Variables in Relation to the Severity of PD

Next, we investigated the clinical periodontal characteristics stratified into two groups based on PD stages II and III/IV. Periodontal variables including PI, BOP, PPD, CAL, number of missing teeth, mobility, and furcation involved teeth as well as stimulated salivary rate flow, collected during the clinical dental examination, are demonstrated in Table 2. In the group with stage III/IV, an increase (*p* < 0.05) was found in sites with PPD ≥4 mm with BOP, PPD 4-5 mm and PPD > 5 mm, interproximal sites with PPD ≥5 mm, CAL 3-4 mm, CAL ≥5 mm, as well as interproximal sites with CAL ≥4 mm, and CAL ≥6 mm, number of missing teeth, and furcation involvements, as compared to group stage II. The presence of CAL 1-2 mm, however, was higher (*p*= 1.34 × 10^−6^) in PD stage II as compared to PD stage III/IV. No significant differences could be observed in PI, BOP, stimulated salivary flow rate, or total protein concentrations when comparing the two groups.

### 3.3. Levels of Inflammatory Mediators in Saliva Samples

Levels of numerous inflammatory mediators, included in the human inflammation panel, were analyzed in stimulated saliva samples. Among the analyzed inflammatory mediators (Table 3), salivary levels of APRIL/TNFSF13, BAFF/TNFSF13B, and IFN-*α*2 were significantly higher (*p* < 0.05) in the group with a severe form of PD (stage III/IV) compared to the group with PD stage II (Table 3, Supporting Information 1: Figure S1A). The remaining inflammatory mediators were not significant (*p* > 0.05) and are listed in Table 3.

### 3.4. Levels of Inflammatory Mediators in Serum Samples

Levels of corresponding inflammatory mediators determined in serum samples of participants, with different stages of PD, are presented in Table 4. When comparing PD stage II with PD stage III/IV, levels of APRIL/TNFSF13, sCD30/TNFRSF8, IL-10, IL-19, and osteopontin were higher (*p* < 0.05) in the PD group with stage III/IV (Table 4, Supporting Information 1: Figure S1B). The inflammatory mediators in serum that were not statistically significant are presented in Table 4.

### 3.5. Relation of PD Severity with Levels of Salivary and Serum Inflammatory Mediators

Next, we performed logistic regression analysis including salivary and serum inflammatory mediators as independent variables, adjusted for age, ACPA, and smoking (former/current smokers) (Tables 5 and 6). None of the potential confounders were included in the models (*p* > 0.10). Concerning salivary mediators, when using PPD ≥4 mm and BOP as the dependent variable, the cytokines APRIL/TNFSF13, IL-34, sTNF-R2, and TWEAK/TNFSF12 were positively correlated with PPD ≥4 mm and BOP (Table 5). Furthermore, when using BI as the dependent variable (Table 5), the highest OR among salivary inflammatory mediators was found for BAFF/TNFSF13B and sTNF-R2 both showing a positive correlation with BI. On the contrary, the inflammatory mediators sCD163, IL-19, and MMP-3 were negatively correlated with BI. Moreover, when using stage III/IV vs. stage II as the dependent variable, positive correlations were found for salivary IFN-*α*2 levels and age (Table 5). Overall, for the logistic regression analyses using the three periodontal parameters: PPD ≥4 mm and BOP, BI, and stage as the dependent variable; 83%, 81%, and 80%, respectively, of the cases were correctly classified by the model.

With regard to serum samples, using PPD ≥4 mm and BOP as the dependent variable, a positive correlation was found with the levels of Gp130/sIL-6Rb (Table 6). Additionally, using BI as the dependent variable, former/current smoking status showed a negative correlation (Table 6). When employing stage III/IV vs. stage II as the dependent variable, a positive correlation was observed between the levels of IL-19 and age. ACPA positivity was shown to be negatively correlated with stage III/IV. In the logistic regression analyses using PPD ≥4 mm and BOP, BI, and PD stages as the dependent variables, the model correctly classified 69%, 66%, and 73% of the cases, respectively.

### 3.6. Characterization of Similarities and Differences between PD Stage II and Stage III/IV

**Figure 1:**
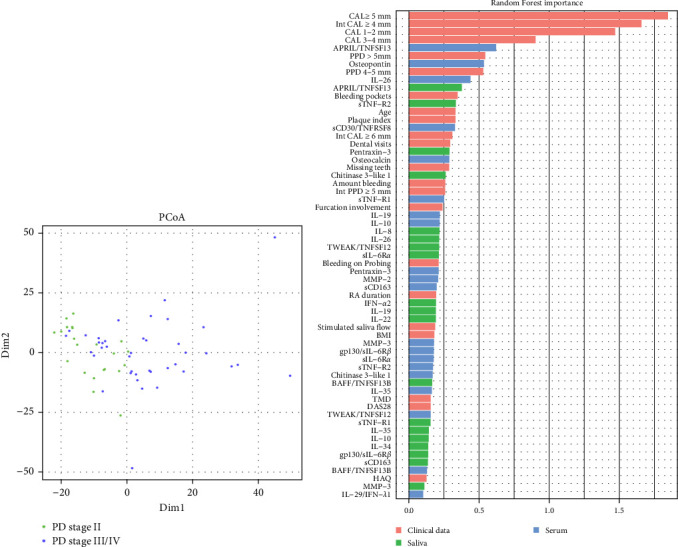
(A) Principal coordinate analysis (PCoA) plots, based on Manhattan distances, illustrating the included samples color-coded by periodontal clinical status, PD stage II and PD stage III/IV. (B) Random Forest feature importance based on MeanDecreaseGini values (*X*-axis). The classification random forest model was trained using periodontal variables (clinical data in red), and inflammatory mediators in saliva (green) and serum (blue) from patients with RA, to classify between patients with PD stage II and stage III/IV. APRIL/TNFSF13, a proliferation-inducing ligand; BAFF/TNFSF13B, B-cell activating factor; BMI, body mass index; CAL, clinical attachment loss; DAS28, disease activity score; Gp130/sIL-6R*β*, glycoprotein 130; HAQ, health assessment questionnaire for rheumatoid arthritis; IFN-*α*2, interferon alpha 2; IL, interleukin; Int, interproximal; MMP, matrix metalloproteinase; PD, periodontitis; PPD, probing pocket depth; RA, rheumatoid arthritis; sCD163, soluble cluster of differentiation 163; sCD30/TNFSF8, soluble CD30; sIL-6R*α*, soluble interleukin-6 receptor alpha; sTNF-R1, soluble TNF receptor 1; sTNF-R2, soluble TNF receptor 2; TMD, disorders involving the temporomandibular joint; TWEAK/TNFSF12, tumor necrosis factor-like weak inducer of apoptosis.

To visualize similarities and dissimilarities between the group with PD stage II and stage III/IV in patients with RA, PCoA was performed based on Manhattan distances (Figure 1A). Trends leaned toward the left side for the group with PD stage II, while the group with PD stages III/IV showed less uniformity. To further investigate whether the collected clinical data and salivary and/or serum inflammatory mediators could be used to distinguish between PD stage II and stage III/IV, we applied a Random Forest model (Figure 1B). The dataset was split (stratified sampling), where 46 samples were used to train the model and 16 samples to test the model. The analysis revealed the periodontal variables: CAL ≥5 mm, intCAL ≥4 mm, CAL 1-2 mm, CAL 3-4 mm, and PPD >5 mm and the serum inflammatory mediators APRIL/TNFSF13 and osteopontin as important variables (MeanDecreaseGini values >0.5) distinguishing between PD stage II and PD stage III/IV in patients with RA. The OOB approach on train dataset (46 samples) showed an F1 score of 0.852, and the model validation using the test dataset (16 samples), showed an F1 score of 0.737 and an AUC of 0.85.

### 3.7. Correlations between Salivary and Serum Inflammatory Mediators

**Figure 2:**
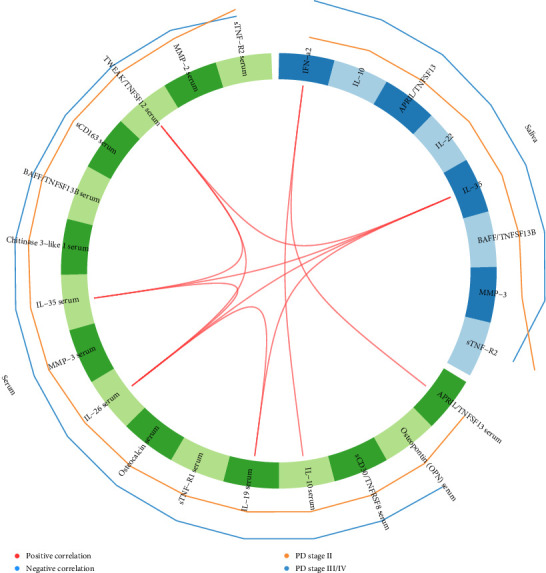
Circos plot illustrating inter- and intra-correlations between salivary and serum inflammatory mediators. Blue and green variables represent inflammatory mediators in saliva and serum, respectively. Red lines within the circle indicate positive correlations between inflammatory mediators at a correlation cutoff of 0.75. Orange and blue lines outside the circle represent patients with periodontitis (PD) stage II and stage III/IV, respectively. APRIL/TNFSF13, a proliferation-inducing ligand; BAFF/TNFSF13B, B-cell activating factor; IFN-*α*2, interferon alpha 2; IL, interleukin; MMP, matrix metalloproteinase; sCD163, soluble cluster of differentiation 163; sCD30/TNFSF8, soluble CD30; sTNF-R1, soluble TNF receptor 1; sTNF-R2, soluble TNF receptor 2; TWEAK/TNFSF12, tumor necrosis factor-like weak inducer of apoptosis.

Figure 2 shows a Circos plot illustrating inter- and intracorrelations between salivary and serum inflammatory mediators. Positive correlations were observed between salivary IFN-*α*2 and serum levels of APRIL/TNFSF13 and IL-10, as well as between salivary IL-35 and serum TWEAK/TNFSF12, IL-35, IL-26, and IL-19. Additionally, positive intracorrelations were identified in serum between IL-26 and TWEAK/TNFSF12, IL-35 and IL-19, as well as between TWEAK/TNFSF12 and IL-35 (Figure 2).

## 4. Discussion

The objectives of this study were to investigate periodontal status based on the current periodontal classification and assess the levels of local and systemic inflammatory mediators in patients with RA. Our findings demonstrate a high prevalence of PD stage III/IV in patients with RA and elevated levels of the cytokine APRIL/TNFSF13 in both saliva and serum of RA subjects with severe PD (stage III/IV).

In our RA cohort, 100% of the participants exhibited clinical signs of periodontal disease (PD stage II–IV). Previous studies investigating the prevalence of PD in patients with RA have reported an increased risk and higher prevalence of severe PD when compared to non-RA controls [21, 24, 25]. Research on the prevalence of PD in patients with RA, in relation to the current AAP/EFP periodontal classification scheme, is scarce. In a previous study, our research group reported that 75% of the RA participants exhibited moderate to severe PD, whereas the remaining individuals exhibited no or mild PD, based on the former PD classification criteria from the year 2007 [27]. Regarding the general prevalence of PD using the current periodontal classifications scheme, a study investigated the prevalence of PD in a Norwegian population. Based on their findings, the prevalence of PD in adults was 72.4%, whereas 41% was diagnosed with PD stage II and 17.5% with stage III/IV [2]. Findings from a Swedish study that used similar diagnostic criteria for PD revealed a 40% prevalence of moderate and severe PD [32]. Furthermore, a recent global study reported that 12.5% of individuals had severe PD [33]. In this study, 64.5% had more severe PD (stage III/IV), which suggests that patients diagnosed with RA have a high prevalence of severe PD. The variation in PD prevalence may be influenced by the periodontal classification systems and employed case definitions used [34, 35] potentially explaining the observed discrepancies. In addition, the prevalence of PD can vary significantly based on factors such as age, geographic location, ethnicity, educational level, availability of health services, and systemic health [36, 37].

In chronic inflammatory diseases, such as PD and RA, inflammatory mediators play an important role in promoting and maintaining the state of chronic inflammation, eventually leading to the destruction of tissue and bone [5, 38]. The cytokine APRIL/TNFSF13, from the TNF superfamily, is a proliferation ligand known to be involved in autoimmune diseases including RA [39, 40]. In our study, both salivary and serum levels of APRIL/TNFSF13 were higher in RA patients with severe periodontal disease (PD stage III/IV) as compared to PD stage II. In addition, our results showed that salivary levels of APRIL/TNFSF13 were positively correlated with inflamed periodontal pockets. Salivary levels of BAFF/TNFSF13B, another member of the TNF superfamily, were also higher in RA patients with PD stage III/IV compared to stage II as well as positively correlated with BI. Interestingly, in non-RA patients with PD, the levels of APRIL/TNFSF13 and BAFF/TNFSF13B were found to be increased in periodontal lesions and suggested to contribute to periodontal bone destruction [41]. Moreover, in line with our results, increased serum levels of APRIL/TNFSF13 and BAFF/TNFSF13B have been observed in patients with RA and PD compared to non-RA individuals with PD [39]. Members of the TNF superfamily play a crucial role in mediating inflammation, partly by affecting B-cell survival and activation of signal pathways [42]. In RA, B-cells can give rise to the production of autoantibodies such as RF and ACPAs and contribute to RA pathogenesis by the production of different cytokines and chemokines [42]. Similarly in PD, BAFF/TNFSF13B contributes to B-cell activation and survival during periodontal inflammation [43]. Notably, levels of both APRIL/TNFSF13 and BAFF/TNFSF13B are reported to be higher in serum samples of patients with RA and PD compared to non-RA with PD [39]. Serum levels of APRIL/TNFSF13 correlated with salivary levels of IFN-*α*2, which was also significantly correlated with PD stage III/IV in the logistic regression analyses. Taken together, it is plausible that these cytokines, by activation of B-cells, may contribute to elevated levels of inflammatory mediators, sustaining periodontal inflammation and leading to severe PD in patients with RA.

Regarding other cytokines within the TNF superfamily, salivary levels of sTNF-R2 and TWEAK/TNFSF12 were positively correlated with periodontal inflammation in terms of inflamed periodontal pockets. Additionally, salivary sTNF-R2 levels were also correlated with BI and may be of importance in distinguishing between PD stage III/IV and stage II in patients with RA. In line with our findings, a study conducted by Kibune et al. [44] demonstrated an association between elevated levels of salivary sTNF-R2 and periodontal inflammation. Elevated levels of TWEAK/TNFSF12 have been suggested to be related to the exacerbation of periodontal diseases and contribute to RA pathogenesis by inducing the production of proinflammatory cytokines [45]. This cytokine was also observed to be significantly higher in patients with PD and RA in saliva samples, compared to patients with PD without RA [46]. Moreover, the soluble TNF-*α* receptors, sTNF-R1, and sTNF-R2 have been suggested to be involved in modulating and balancing TNF-*α* activity in inflammation [47].

In serum, patients with PD stage III/IV exhibited elevated levels of the cytokines: sCD30, IL-10, IL-19, and osteopontin, as compared to stage II. Moreover, serum levels of osteopontin may also be important for classifying between PD stage III/IV and stage II. Osteopontin, an inflammatory glycoprotein associated with bone resorption [48], has previously been reported to be increased in both plasma and gingival crevicular fluid (GCF) samples from sites with periodontal destruction [49], indicating its association with aggravated periodontal disease, further supporting the results from this study. Regarding the cytokine IL-19, our research group has previously reported higher levels of IL-19 in GCF of patients with RA and moderate/severe PD compared to RA subjects with no/mild PD [27]. These findings are consistent with current results showing increased levels of IL-19 in PD stage III/IV. Interestingly, IL-19 has also been suggested to possess anti-inflammatory properties in RA [50], which is in line with the negative correlation between salivary IL-19 levels and BI observed in this study. Thus, IL-19 may play a complex role in the immune system, and further studies are needed to clarify its role in patients with PD and RA. Like IL-19, the cytokine IL-10 also has anti-inflammatory properties and inhibits inflammation, autoreactivity, and production of pro-inflammatory cytokines [51]. Serum levels of IL-10 have previously been shown to be increased in patients with RA, and prior research suggests that IL-10 may play a contradictory role in RA by enhancing the humoral autoimmune response [52, 53]. This dual function may explain the elevated serum levels of IL-10 observed in this study. While IL-10 is primarily known for its anti-inflammatory properties, IL-6 is known to be pro-inflammatory and has a crucial role in modulating immune responses and inflammation. The soluble forms of the IL-6 receptor, sIL6R*α*, and gp130/sIL-6R*β* are suggested to play an important role in the IL-6 signaling pathway, potentially contributing to the inflammatory cascade observed in chronic inflammatory diseases such as PD and RA [54]. The aforementioned is in line with the results from the current study, demonstrating that serum levels of gp130/sIL-6R*β* are correlated with inflamed periodontal pockets.

In this study, salivary levels of IL-34 were negatively associated with inflamed periodontal pockets (PPD ≥4 mm and BOP). In line with our results, salivary levels of IL-34 have previously been demonstrated to be associated with periodontal health and negatively correlated with parameters of periodontal inflammation such as PPD and BOP [55]. However, levels of IL-34 in serum and GCF samples have been shown to be increased in patients with chronic PD, potentially contributing to alveolar bone loss in periodontal lesions [56]. These findings suggest that IL-34 expression may vary between saliva, GCF, and serum samples.

As expected, former/current smoking habits were negatively correlated with BI, corresponding to previous studies suggesting a strong association with reduced BI in smokers [57]. Additionally, the prevalence of former/current smokers was higher in the group with PD stage III/IV compared to stage II, which is consistent with the well-established association between smoking and increased risk for both PD and RA [58]. Furthermore, a higher prevalence of PD has been demonstrated in current smokers with ACPA-positive RA [59]. In the current study, ACPA positivity negatively correlated with PD stage III/IV, and no medication differences were observed between ACPA-positive and ACPA-negative patients (Supporting Information 2: Table S1). Nevertheless, recent findings indicate that a subset of ACPA might have protective potential in RA [60, 61], highlighting its multifaceted role in disease management and progression.

One of the study's strengths was the use of both saliva and serum samples from each participant. This dual-sample analyzing methodology enhanced our findings to compare potential biomarkers locally and systemically. This approach provided a better understanding of the interactions occurring in the mouth and throughout the body in patients with PD and RA. Additionally, one single dentist examined all participants excluding interexaminer variability. Another strength of this study was the confirmation of well-known risk factors (former/current smoking habits). This study also has some limitations. Since RA is more common in females compared to males [12], we were not able to match the groups for gender which resulted in a higher prevalence of females compared to males. Additionally, incorporating a comparison group with PD but without RA could have provided further insights. However, our group addressed this in a previous study, comparing salivary inflammatory mediators in individuals with chronic PD, with or without RA. In that study, we reported that TWEAK/TNFSF12, IFN-*α*2, and IL-19 were higher in saliva samples from patients with both PD and RA compared to those with PD alone [46]. Moreover, some participants had systemic diseases such as diabetes and cardiovascular conditions that are suggested to be associated with PD. However, no significant differences were found regarding systemic diseases between the groups. Another limitation of this study is the relatively small sample size of 62 participants. While our power analysis estimated a minimum of 59 samples, the cohort size may still limit the generalizability of the findings in our well-characterized cohort.

To our knowledge, this is the first study to investigate the prevalence of PD using the current periodontal classification scheme and compare salivary and serum inflammatory mediators in relation to periodontal stages in patients with RA. Our findings revealed several significantly higher inflammatory mediators in both saliva and serum when comparing PD severity within the RA cohort. These results suggest that PD may contribute to increased systemic inflammation in RA, as indicated by higher serum inflammatory mediator levels in patients with severe PD (stage III/IV) compared to those with moderate PD (stage II).

## 5. Conclusion

In this cohort of RA patients, the majority were diagnosed with severe PD (stage III/IV), highlighting the necessity of periodontal prophylaxis and treatment for this group of patients. Elevated levels of inflammatory mediators were detected in both saliva and serum in patients with severe PD, suggesting a potential relationship between the severity of periodontal disease and increased systemic inflammation in RA. Further research is required to evaluate the clinical significance of these findings and the potential benefits of early intervention.

## Figures and Tables

**Table 1:** Characteristics of all the study population (*n* = 62) and divided into two groups: periodontitis stage II (*n* = 22) and periodontitis stage III/IV (*n* = 40).

Characteristics	All study participants (*n* = 62)	Periodontitis Stage II (*n* = 22)	Periodontitis stage III/IV (*n* = 40)	*p⁣* ^*∗*^
Female gender, *n* (%)	56 (90.3)	22 (100)	34 (85)	0.14
Age, median (Q25%; Q75%)	64 (55; 70)	59 (41; 69)	66 (62; 71)	0.021
BMI, median (Q25%; Q75%)	24.2 (20.8; 27.8)	24.7 (20.7; 28.3)	23.6 (20.7; 27.6)	0.94
RA duration (years) median (Q25%; Q75%)	8.5 (2.6; 16.0)	10.0 (3.5; 16.0)	8.0 (2.0; 16.7)	0.61
Comorbidities, *n* (%)				
Diabetes	4 (6.5)	1 (4.5)	3 (7.5)	0.67
Cardiac disorders	8 (12.9)	3 (13.5)	5 (12.5)	0.91
Vascular disorders	5 (8.1)	1 (4.5)	4 (10)	0.79
High blood pressure	12 (19.4)	4 (18.2)	8 (20)	0.87
Gastrointestinal disorders	13 (21.0)	2 (9.1)	11 (27.5)	0.19
Osteoporosis	5 (8.1)	2 (9.1)	3 (7.5)	0.84
Asthma	13 (20.1)	3 (13.6)	10 (25)	0.52
Sjögrens syndrome	6 (10.0)	3 (13.6)	3 (7.5)	0.74
Liver disease	3 (4.8)	0 (0)	3 (7.5)	0.48
TMD	25 (40.3)	13 (59.1)	12 (30)	0.049
Medication, *n* (%)				
Analgesics/NSAID	31 (50)	11 (50)	20 (50)	0.88
DMARDs	42 (67.7)	15 (68.2)	27 (67.5)	0.96
bDMARDs	24 (38.7)	10 (45.5)	14 (35)	0.59
Glucocorticoids	28 (45.2)	8 (36.3)	20 (50)	0.44
Current/former smokers, *n* (%)	41 (66.1)	10 (45.5)	40 (77.5)	0.010
Current snuff, *n* (%)	6 (9.7)	0 (0)	6 (15)	0.14
Alcohol consumption, *n* (%)				
Monthly	37 (59.7)	13 (59.1)	24 (60)	0.80
Weekly	32 (51.6)	10 (45.5)	22 (55)	0.52
Daily	7 (11.3)	1 (4.5)	6 (15)	0.41
University degree, *n* (%)	27 (43.5)	11 (50)	16 (40)	0.30
ACPA positive, *n* (%)	48 (77.4)	18 (81.2)	30 (75)	0.55
DAS-28, median (Q25%; Q75%)	3.3 (2.4; 4.1)	3.1 (2.1; 3.9)	3.3 (2.5; 4.3)	0.44
RF (IU/mL) positive, *n* (%)	40 (64.5)	12 (54.5)	28 (70)	0.62
HAQ score, median (Q25%; Q75%)	0.88 (0.38; 1.4)	0.69 (0.25; 1.2)	1.0 (0.5; 1.5)	0.20
CRP (ng/mL), median (Q25%; Q75%)	3 (1; 5)	2 (1; 5)	3 (1; 5)	0.34

*Note:* Data are presented as median (Q25%; Q75%) or as number (percentage).

Abbreviations: ACPA, anticitrullinated peptide antibodies; bDMARDs, biological disease modifying antirheumatic drugs; BMI, body mass index; CRP, C-reactive protein; DAS-28, disease activity score; DMARDs, disease-modifying antirheumatic drugs; HAQ, health assessment questionnaire for rheumatoid arthritis; NSAID, nonsteroidal anti-inflammatory drugs; RA, rheumatoid arthritis; RF, rheumatoid factor; TMD, disorders involving the temporomandibular joint.

*⁣* ^*∗*^Comparison between groups with periodontitis stage II and stage III/IV.

**Table 2:** Periodontal characteristics divided into two groups in relation to periodontal classification: periodontitis stage II (*n* = 22) and periodontitis stage III/IV (*n* = 40).

Periodontal variables, median (Q25%; Q75%)	Periodontitis stage II (*n* = 22)	Periodontitis stage III/IV (*n* = 40)	*p⁣* ^*∗*^
Plaque Index	52.2 (42.4; 68.2)	47.3 (34.8; 62.5)	0.12
Bleeding Index	27.7 (16.2; 60.9)	32.9 (22.7; 49.2)	0.98
Sites with PPD ≥ 4 mm and BOP	1.2 (0.60; 3.3)	3.9 (1.3; 8.8)	0.0031
Sites with PPD 4–5 mm	1.2 (0.60; 4.2)	6.7 (2.3; 12.2)	0.00047
Sites with PPD >5 mm	0.0 (0.0; 0.0)	0.70 (0.0; 2.4)	4.43e–05
Interproximal sites with PPD ≥5 mm	0.0 (0.0; 0.25)	2.0 (0.0; 4.0)	0.00065
Sites with CAL			
1–2 mm	71.5 (59.4; 84.1)	35.2 (15.9; 58.9)	1.34e–06
3–4 mm	25.1 (10.0; 25.1)	54.3 (31.5; 66.1)	1.16e–05
≥5 mm	0.0 (0.0; 0.85)	5.1 (2.4; 14.8)	1.75e–07
Interproximal sites with CAL			
≥4 mm^†^	1.0 (0.0; 2.2)	6.0 (4.0; 14.7)	7.92e–07
≥6 mm	0.0 (0.0; 0.0)	0.0 (0.0; 3.0)	0.00017
Missing teeth	3.6 (0.0; 7.1)	10.7 (3.6; 20.5)	0.0028
Mobile teeth	0.0 (0.0; 0.0)	0.0 (0.0; 13.9)	0.14
Furcation involvement	6.4 (2.7; 21.4)	17.0 (10.3; 23.5)	0.016
Stimulated salivary flow rate (ml/min)	1.7 (1.2; 2.3)	1.5 (0.72; 2.0)	0.29
Total salivary protein (mg/ml)	1.8 (1.6; 2.5)	2,0 (1.5; 2.7)	0.42

*Note:* Data are presented as median (Q25%; Q75%) or as number (percentage).

Abbreviations: BOP, bleeding on probing; CAL, clinical attachment loss; PPD, probing pocket depth.

*⁣* ^*∗*^Comparison between groups with periodontitis stage II and stage III/IV.

^†^For periodontitis stage II, interproximal sites with CAL did not exceed 4 mm.

**Table 3:** Levels of inflammatory mediators (pg/ml) in saliva in relation to periodontal classification: periodontitis stage II (*n* = 22) and periodontitis stage III/IV (*n* = 40).

Inflammatory mediators in saliva, median (Q25%; Q75%)	Periodontitis stage II (*n* = 22)	Periodontitis stage III/IV (*n* = 40)	*p⁣* ^*∗*^
APRIL/TNFSF13	33,572 (15215; 61750)	60,643 (34359; 175252)	0.018
BAFF/TNFSF13B	2072 (1561; 3088)	2995 (2306; 4146)	0.039
sCD163	731 (17; 1676)	927 (17; 2502)	0.60
Chitinase 3-like 1	6287 (3488; 13861)	6576 (3261; 11011)	0.53
gp130/sIL-6R*β*	7958 (1636; 10397)	6767 (2773; 12948)	0.63
IFN-*α*2	28 (8.1; 73)	59 (33; 112)	0.019
sIL-6R*α*	151 (54; 471)	238 (96; 571)	0.38
IL-8	750 (453; 1418)	893 (416; 1451)	0.71
IL-10	2.5 (0.82; 5.2)	3.8 (2.1; 6.5)	0.067
IL-19	86 (53; 263)	177 (45; 338)	0.45
IL-22	6.2 (1.1; 11)	7.9 (2.5; 20)	0.095
IL-26	6.4 (2.6; 8.4)	6.8 (2.7; 15)	0.18
IL-34	125 (52; 377)	226 (58; 449)	0.35
IL-35	123 (63; 231)	187 (91; 340)	0.15
MMP-3	83 (28; 594)	297 (28; 936)	0.33
Pentraxin-3	180 (73; 416)	265 (145; 597)	0.31
sTNF-R1	432 (215; 1033)	672 (288; 1159)	0.44
sTNF-R2	22 (5.7; 884)	62 (23; 310)	0.68
TWEAK/TNFSF12	25 (14; 64)	45 (21; 106)	0.12

*Note:* Data are presented as median (Q25%; Q75%).

Abbreviations: APRIL/TNFSF13, a proliferation-inducing ligand; BAFF/TNFSF13B, B-cell-activating factor; gp130/sIL-6R*β*, glycoprotein 130; IFN-*α*2, interferon alpha 2; IL, interleukin; MMP-3, matrix metalloproteinase-3; sCD163, soluble cluster of differentiation 163; sIL-6R*α*, soluble interleukin-6 receptor alpha; sTNF-R1, soluble TNF receptor 1; sTNF-R2, soluble TNF receptor 2; TWEAK/TNFSF12, tumor necrosis factor-like weak inducer of apoptosis.

*⁣* ^*∗*^Comparison between groups with periodontitis stage II and stage III/IV.

**Table 4:** Inflammatory mediator levels (pg/ml) in serum in relation to periodontal classification: periodontitis stage II (*n* = 22) and periodontitis stage III/IV (*n* = 40).

Inflammatory mediators in serum, median (Q25%; Q75%)	Periodontitis stage II (*n* = 22)	Periodontitis stage III/IV (*n* = 40)	*p⁣* ^*∗*^
APRIL/TNFSF13	62,921 (39482; 103618)	128,109 (65667; 152030)	0.0013
BAFF/TNFSF13B	7828 (5658; 9446)	9675 (6851; 14375)	0.083
sCD30/TNFSF8	176 (133; 321)	266 (197; 419)	0.028
sCD163	46,318 (35479; 65115)	50,938 (36564; 80064)	0.47
Chitinase 3-like 1	12,500 (9148; 17420)	14,823 (8743; 22568)	0.48
Gp130/sIL-6R*β*	27,528 (24303; 32064)	28,855 (23286; 31126)	0.80
sIL-6R*α*	12,302 (9467; 14765	12,729 (10312; 13386)	0.81
IL-10	8.3 (2.6; 18)	17 (11; 19)	0.024
IL-19	9.6 (2.6; 26)	27 (16; 33)	0.0050
IL-26	54 (38; 63)	61 (51; 75)	0.084
IL-29/IFN-*λ*1	14 (7.7; 86)	49 (2.3; 98)	0.58
IL-35	32 (19; 63)	43 (31; 86)	0.17
MMP-2	3731 (3226; 7680)	4716 (3703; 7171)	0.43
MMP-3	4611 (3468; 9286)	6577 (3967; 10312)	0.18
Osteocalcin	1557 (937; 1710)	1588 (1106; 2294)	0.14
Osteopontin	18,284 (14434; 23828)	27,096 (20544; 34139)	0.014
Pentraxin-3	176 (87; 224)	192 (156; 289)	0.095
sTNF-R1	1817 (1280; 2482)	2243 (1902; 3069)	0.079
sTNF-R2	2,172 (1402; 3934)	2361 (1497; 5049)	0.65
TWEAK/TNFSF12	161 (133; 184)	167 (139; 198)	0.64

*Note:* Data are presented as median (Q25%; Q75%).

Abbreviations: APRIL/TNFSF13, a proliferation-inducing ligand; BAFF/TNFSF13B, B-cell-activating factor; Gp130/sIL-6R*β*, glycoprotein 130; IL, interleukin; MMP, matrix metalloproteinase; sCD163, soluble cluster of differentiation 163; sCD30/TNFSF8, soluble CD30; sIL-6R*α*, soluble interleukin-6 receptor alpha; sTNF-R1, soluble TNF receptor 1; sTNF-R2, soluble TNF receptor 2; TWEAK/TNFSF12, tumor necrosis factor-like weak inducer of apoptosis.

*⁣* ^*∗*^Comparison between groups with periodontitis stage II and stage III/IV.

**Table 5:** Logistic regression analysis using bleeding pockets (sites with PPD ≥4 mm and BOP ≤3.0, >3.0), bleeding index (≤30, >30), and PD stage III/IV vs. stage II as the dependent variables and inflammatory mediators in saliva and potential confounders as independent variables.

Dependent variables	Independent variables	OR	95% CI	*p*
PPD ≥4 mm and BOP	APRIL/TNFSF13	1.71	1.3–2.85	0.039
	IL-10	2.58	0.96–6.95	0.060
	IL-34	0.12	0.03–0.49	0.003
	sTNF-R2	1.58	1.01–2.48	0.047
	TWEAK/TNFSF12	3.55	1.11–11.33	0.032
Bleeding index	BAFF/TNFSF13B	10.71	1.33–85.95	0.026
	sCD163	0.59	0.35–0.98	0.041
	IL-10	2.42	0.98–6.02	0.056
	IL-19	0.39	0.18–0.85	0.017
	MMP-3	0.51	0.27–0.97	0.039
	sTNF-R2	1.97	1.14–3.38	0.015
Stage III/IV vs. stage II	Age	1.07	1.01–1.14	0.033
	IFN-*α*2	1.75	1.01–3.03	0.046

*Note:* The results were adjusted for age, ACPA status, and former/current smokers. Odds ratios (OR) with 95% confidence intervals (CI) are presented.

Abbreviations: ACPA, anti-citrullinated protein antibody; APRIL/TNFSF13, a proliferation-inducing ligand; BAFF/TNFSF13B, B-cell-activating factor; BOP, bleeding on probing; IFN-*α*2, interferon alpha 2; IL, interleukin; MMP-3, matrix metalloproteinase 3; PD, periodontitis; PPD, probing pocket depth; sCD163, soluble cluster of differentiation 163; sTNF-R2, soluble TNF receptor 2; TWEAK/TNFSF12, tumor necrosis factor-like weak inducer of apoptosis.

**Table 6:** Logistic regression analysis using bleeding pockets (sites with PPD ≥4 mm and BOP ≤3.0, >3.0), bleeding index (≤30, >30), and PD stage III/IV vs. stage II as the dependent variables and inflammatory mediators in serum and potential confounders as independent variables.

Dependent variables	Independent variables	OR	95% CI	*p*
PPD ≥4 mm and BOP	Gp130/sIL-6R*β*	34.09	2.11–549.63	0.013
	IL-26	4.93	0.75–32.26	0.096
Bleeding index	Former/current smokers	0.21	0.05–0.92	0.038
	IL-19	1.96	0.96–4.03	0.065
	sCD163	0.30	0.07–1.17	0.083
Stage III/IV vs. stage II	Age	1.11	1.02–1.20	0.013
	ACPA positive	0.07	0.01–0.74	0.027
	IL-19	3.00	1.37–6.57	0.006

*Note:* The results were adjusted for age, ACPA status, and former/current smokers. Odds ratios (OR) with 95% confidence intervals (CI) are presented.

Abbreviations: ACPA, anti-citrullinated protein antibody; BOP, bleeding on probing; Gp130/sIL-6R*β*, glycoprotein 130; IL, interleukin; PD, periodontitis; PPD, probing pocket depth; sCD163, soluble cluster of differentiation 163.

## Data Availability

The data that support the findings of this study are available from the corresponding author upon reasonable request.

## Supporting Information

Supporting Information 1 Figure S1: Levels of inflammatory mediators in saliva (A) and serum samples (B), when comparing periodontitis stage II with stage III/IV. APRIL/TNFSF13, a proliferation-inducing ligand; BAFF/TNFSF13B, B-cell activating factor; IFN-*α*2, interferon alpha 2; IL, interleukin; PD, periodontitis; sCD30/TNFSF8, soluble CD30.

Supporting Information 2 Table S1: Medication of all participants, divided into two groups, ACPA-positive and ACPA-negative.

## References

[1] Eke P. I., Thornton-Evans G. O., Wei L., Borgnakke W. S., Dye B. A., Genco R. J. (2018). Periodontitis in US Adults: National Health and Nutrition Examination Survey 2009–2014. *Journal of the American Dental Association*.

[2] Stodle I. H., Verket A., Hovik H., Sen A., Koldsland O. C. (2021). Prevalence of Periodontitis Based on the 2017 Classification in a Norwegian Population: The HUNT Study. *Journal of Clinical Periodontology*.

[3] Hajishengallis G., Liang S., Payne M. A. (2011). Low-Abundance Biofilm Species Orchestrates Inflammatory Periodontal Disease Through the Commensal Microbiota and Complement. *Cell Host & Microbe*.

[4] Bascones A., Noronha S., Gomez M., Mota P., Gonzalez Moles M. A., Villarroel Dorrego M. (2005). Tissue Destruction in Periodontitis: Bacteria or Cytokines Fault?. *Quintessence International*.

[5] Yucel-Lindberg T., Bage T. (2013). Inflammatory Mediators in the Pathogenesis of Periodontitis. *Expert Reviews in Molecular Medicine*.

[6] Page R. C., Kornman K. S. (1997). The Pathogenesis of Human Periodontitis: An Introduction. *Periodontology 2000*.

[7] Graves D. T., Cochran D. (2003). The Contribution of Interleukin-1 and Tumor Necrosis Factor to Periodontal Tissue Destruction. *Journal of Periodontology*.

[8] Ramadan D. E., Hariyani N., Indrawati R., Ridwan R. D., Diyatri I. (2020). Cytokines and Chemokines in Periodontitis. *European Journal of Dentistry*.

[9] Genco R., Offenbacher S., Beck J. (2002). Periodontal Disease and Cardiovascular Disease: Epidemiology and Possible Mechanisms. *The Journal of the American Dental Association*.

[10] Taylor G. W. (2001). Bidirectional Interrelationships Between Diabetes and Periodontal Diseases: An Epidemiologic Perspective. *Annals of Periodontology*.

[11] Mercado F., Marshall R. I., Klestov A. C., Bartold P. M. (2000). Is There a Relationship Between Rheumatoid Arthritis and Periodontal Disease?. *Journal of Clinical Periodontology*.

[12] Klareskog L., Catrina A. I., Paget S. (2009). Rheumatoid Arthritis. *The Lancet*.

[13] Smolen J. S., Aletaha D., McInnes I. B. (2016). Rheumatoid Arthritis. *The Lancet*.

[14] Davison E., Johnston W., Piela K. (2021). The Subgingival Plaque Microbiome, Systemic Antibodies Against Bacteria and Citrullinated Proteins Following Periodontal Therapy. *Pathogens*.

[15] de Molon R. S., Rossa C., Thurlings R. M., Cirelli J. A., Koenders M. I. (2019). Linkage of Periodontitis and Rheumatoid Arthritis: Current Evidence and Potential Biological Interactions. *International Journal of Molecular Sciences*.

[16] Rosenstein E. D., Greenwald R. A., Kushner L. J., Weissmann G. (2004). Hypothesis: The Humoral Immune Response to Oral Bacteria Provides a Stimulus for the Development of Rheumatoid Arthritis. *Inflammation*.

[17] Potempa J., Mydel P., Koziel J. (2017). The Case for Periodontitis in the Pathogenesis of Rheumatoid Arthritis. *Nature Reviews Rheumatology*.

[18] Wegner N., Wait R., Sroka A. (2010). Peptidylarginine Deiminase from *Porphyromonas gingivalis* Citrullinates Human Fibrinogen and Alpha-Enolase: Implications for Autoimmunity in Rheumatoid Arthritis. *Arthritis and Rheumatism*.

[19] Mercado F. B., Marshall R. I., Klestov A. C., Bartold P. M. (2001). Relationship Between Rheumatoid Arthritis and Periodontitis. *Journal of Periodontology*.

[20] Pischon N., Pischon T., Kroger J. (2008). Association Among Rheumatoid Arthritis, Oral Hygiene, and Periodontitis. *Journal of Periodontology*.

[21] Bolstad A. I., Fevang B. S., Lie S. A. (2023). Increased Risk of Periodontitis in Patients With Rheumatoid Arthritis: A Nationwide Register Study in Norway. *Journal of Clinical Periodontology*.

[22] Renvert S., Berglund J. S., Persson G. R., Söderlin M. K. (2020). The Association between Rheumatoid Arthritis and Periodontal Disease in a Population-Based Cross-Sectional Case-Control Study. *BMC Rheumatol*.

[23] Choi Y. Y., Lee K. H. (2021). Periodontitis as a Risk Factor for Rheumatoid Arthritis: A Matched-Cohort Study. *International Dental Journal*.

[24] Karapetsa D., Consensi A., Castagnoli G. (2022). Periodontitis in Italian Patients With Established Rheumatoid Arthritis: A Cross-Sectional Study. *Oral Diseases*.

[25] Punceviciene E., Rovas A., Puriene A. (2021). Investigating the Relationship Between the Severity of Periodontitis and Rheumatoid Arthritis: A Cross-Sectional Study. *Clinical Rheumatology*.

[26] Tonetti M. S., Greenwell H., Kornman K. S. (2018). Staging and Grading of Periodontitis: Framework and Proposal of a New Classification and Case Definition. *Journal of Periodontology*.

[27] Eriksson K., Fei G., Lundmark A. (2019). Periodontal Health and Oral Microbiota in Patients With Rheumatoid Arthritis. *Journal of Clinical Medicine*.

[28] Lundmark A., Johannsen G., Eriksson K. (2017). Mucin 4 and Matrix Metalloproteinase 7 as Novel Salivary Biomarkers for Periodontitis. *Journal of Clinical Periodontology*.

[29] Titusson C., Jansson L., Modin C. (2025). Salivary Inflammatory Mediator Profiles in Periodontal and Peri-Implant Health and Disease: A Cross-Sectional Study. *Clinical Implant Dentistry and Related Research*.

[30] Bylander T. (2002). *Machine Learning*.

[31] Robin X., Turck N., Hainard A. (2011). pROC: An Open-Source Package for R and S+ to Analyze and Compare ROC Curves. *BMC Bioinformatics*.

[32] Wahlin A., Papias A., Jansson H., Norderyd O. (2018). Secular Trends Over 40 Years of Periodontal Health and Disease in Individuals Aged 20–80 Years in Jonkoping, Sweden: Repeated Cross-Sectional Studies. *Journal of Clinical Periodontology*.

[33] Nascimento G. G., Alves-Costa S., Romandini M. (2024). Burden of Severe Periodontitis and Edentulism in 2021, With Projections up to 2050: The Global Burden of Disease 2021 Study. *Journal of Periodontal Research*.

[34] Costa F. O., Guimaraes A. N., Cota L. O. (2009). Impact of Different Periodontitis Case Definitions on Periodontal Research. *Journal of Oral Science*.

[35] Holtfreter B., Albandar J. M., Dietrich T. (2015). Standards for Reporting Chronic Periodontitis Prevalence and Severity in Epidemiologic Studies: Proposed Standards From the Joint EU/USA Periodontal Epidemiology Working Group. *Journal of Clinical Periodontology*.

[36] Eke P. I., Dye B. A., Wei L. (2015). Update on Prevalence of Periodontitis in Adults in the United States: NHANES 2009 to 2012. *Journal of Periodontology*.

[37] Kassebaum N. J., Bernabe E., Dahiya M., Bhandari B., Murray C. J., Marcenes W. (2014). Global Burden of Severe Periodontitis in 1990–2010: A Systematic Review and Meta-Regression. *Journal of Dental Research*.

[38] McInnes I. B., Schett G. (2011). The Pathogenesis of Rheumatoid Arthritis. *The New England Journal of Medicine*.

[39] Gumus P., Buduneli E., Biyikoglu B. (2013). Gingival Crevicular Fluid and Serum Levels of APRIL, BAFF and TNF-Alpha in Rheumatoid Arthritis and Osteoporosis Patients With Periodontal Disease. *Archives of Oral Biology*.

[40] Vincent F. B., Saulep-Easton D., Figgett W. A., Fairfax K. A., Mackay F. (2013). The BAFF/APRIL System: Emerging Functions Beyond B Cell Biology and Autoimmunity. *Cytokine Growth Factor Rev*.

[41] Abe T., AlSarhan M., Benakanakere M. R. (2015). The B Cell-Stimulatory Cytokines BLyS and APRIL are Elevated in Human Periodontitis and are Required for B Cell-Dependent Bone Loss in Experimental Murine Periodontitis. *The Journal of Immunology*.

[42] McInnes I. B., Schett G. (2007). Cytokines in the Pathogenesis of Rheumatoid Arthritis. *Nature Reviews Immunology*.

[43] Dyab A., Emnegard A., Wänman M., Sjöström F., Kindstedt E. (2024). Human Gingival Fibroblasts are a Source of B Cell-Activating Factor During Periodontal Inflammation. *Journal of Periodontology*.

[44] Kibune R., Muraoka K., Morishita M., Ariyoshi W., Awano S. (2022). Relationship Between Dynamics of TNF-*α* and Its Soluble Receptors in Saliva and Periodontal Health State. *Dentistry Journal*.

[45] Hosokawa Y., Hosokawa I., Ozaki K., Nakae H., Matsuo T. (2006). Proinflammatory Effects of Tumour Necrosis Factor-Like Weak Inducer of Apoptosis (TWEAK) on Human Gingival Fibroblasts. *Clinical and Experimental Immunology*.

[46] Eriksson K., Lundmark A., Delgado L. F. (2022). Salivary Microbiota and Host-Inflammatory Responses in Periodontitis Affected Individuals With and Without Rheumatoid Arthritis. *Frontiers in Cellular and Infection Microbiology*.

[47] Tracey K. J., Cerami A. (1994). TUMOR NECROSIS FACTOR: A Pleiotropic Cytokine and Therapuetic Target. *Annual Review of Medicine*.

[48] Ohshima S., Yamaguchi N., Nishioka K. (2002). Enhanced Local Production of Osteopontin in Rheumatoid Joints. *Journal of Rheumatology*.

[49] Sharma C. G., Pradeep A. R. (2007). Plasma and Crevicular Fluid Osteopontin Levels in Periodontal Health and Disease. *Journal of Periodontal Research*.

[50] Kragstrup T. W., Andersen T., Heftdal L. D. (2018). The IL-20 Cytokine Family in Rheumatoid Arthritis and Spondyloarthritis. *Frontiers in Immunology*.

[51] Keystone E., Wherry J., Grint P. (1998). IL-10 as a Therapeutic Strategy in the Treatment of Rheumatoid Arthritis. *Rheumatic Disease Clinics of North America*.

[52] Cush J. J., Splawski J. B., Thomas R. (1995). Elevated Interleukin-10 Levels in Patients With Rheumatoid Arthritis. *Arthritis and Rheumatism*.

[53] Saxena A., Khosraviani S., Noel S., Mohan D., Donner T., Hamad A. R. A. (2015). Interleukin-10 Paradox: A Potent Immunoregulatory Cytokine that Has Been Difficult to Harness for Immunotherapy. *Cytokine*.

[54] Rose-John S. (2012). IL-6 Trans-Signaling via the Soluble IL-6 Receptor: Importance for the pro-Inflammatory Activities of IL-6. *International Journal of Biological Sciences*.

[55] Martinez G. L., Majster M., Bjurshammar N., Johannsen A., Figueredo C. M., Bostrom E. A. (2017). Salivary Colony Stimulating Factor-1 and Interleukin-34 in Periodontal Disease. *Journal of Periodontology*.

[56] Luo Y., Ding Y., Chen Y. (2023). The Role of IL-31 and IL-34 in the Diagnosis and Treatment of Chronic Periodontitis. *Open Life Sciences*.

[57] Al-Bayaty F. H., Baharuddin N., Abdulla M. A., Ali H. M., Arkilla M. B., ALBayaty M. F. (2013). The Influence of Cigarette Smoking on Gingival Bleeding and Serum Concentrations of Haptoglobin and Alpha 1-Antitrypsin. *BioMed research international*.

[58] Vessey M. P., Villard-Mackintosh L., Yeates D. (1987). Oral Contraceptives, Cigarette Smoking and Other Factors in Relation to Arthritis. *Contraception*.

[59] Eriksson K., Nise L., Alfredsson L. (2018). Seropositivity Combined With Smoking Is Associated With Increased Prevalence of Periodontitis in Patients With Rheumatoid Arthritis. *Annals of the Rheumatic Diseases*.

[60] He Y., Ge C., Moreno-Giro A. (2023). A Subset of Antibodies Targeting Citrullinated Proteins Confers Protection From Rheumatoid Arthritis. *Nature Communications*.

[61] He Y., Aoun M., Xu Z., Holmdahl R. (2024). Shift in Perspective: Autoimmunity Protecting Against Rheumatoid Arthritis. *Annals of the Rheumatic Diseases*.

