# Supplementary figures and images for: Comparative Analysis of Salivary and Serum Inflammatory Mediator Profiles in Patients With Rheumatoid Arthritis and Periodontitis
Source: Mediators Inflamm. 2025 Mar 20;2025:7739833. doi: 10.1155/mi/7739833 (PMC11949604; doi:10.1155/mi/7739833)

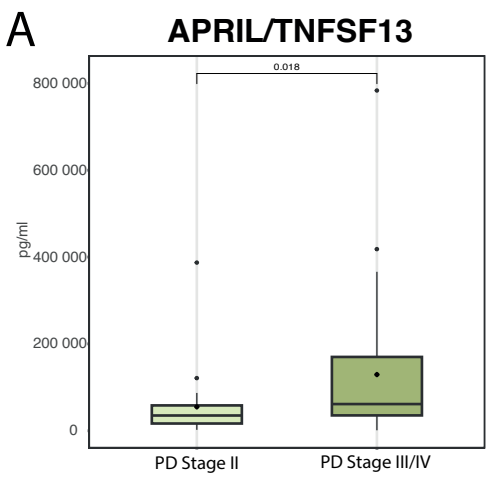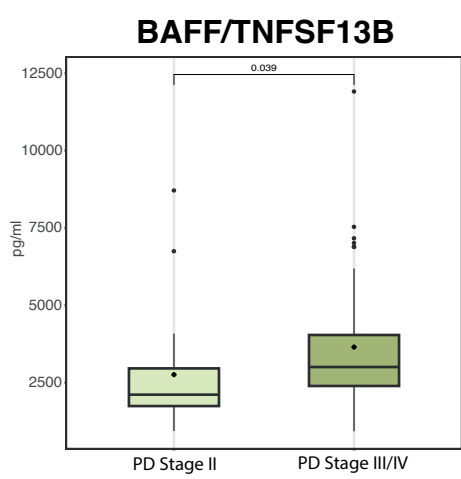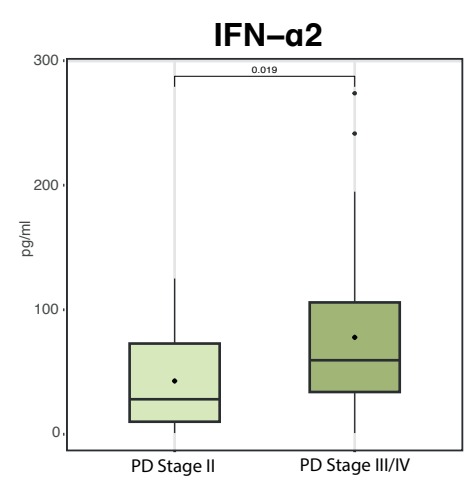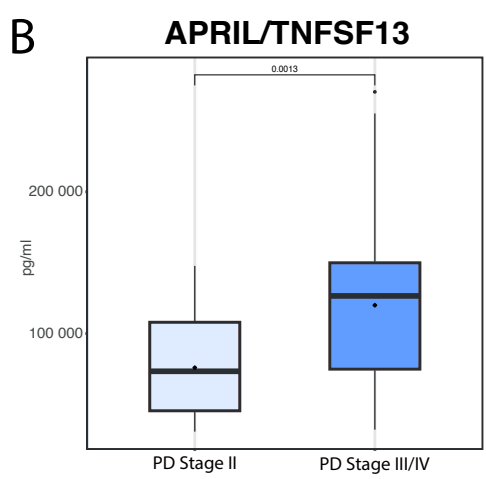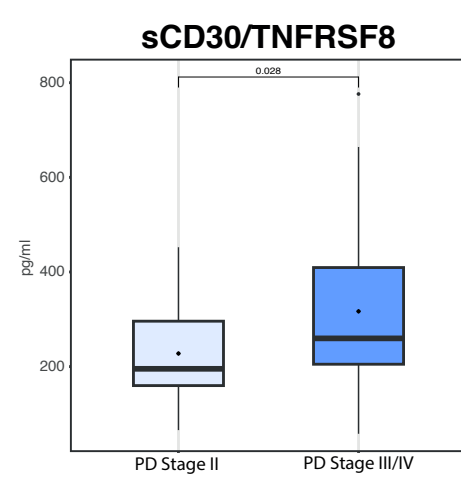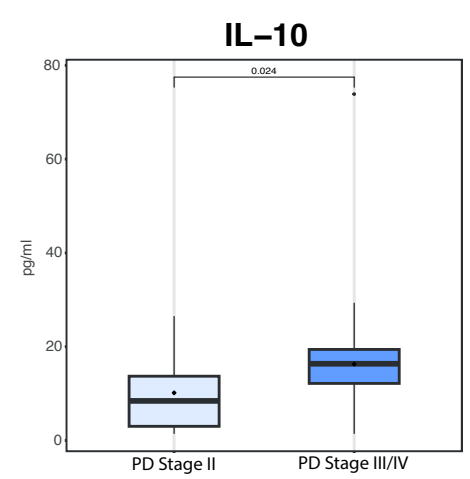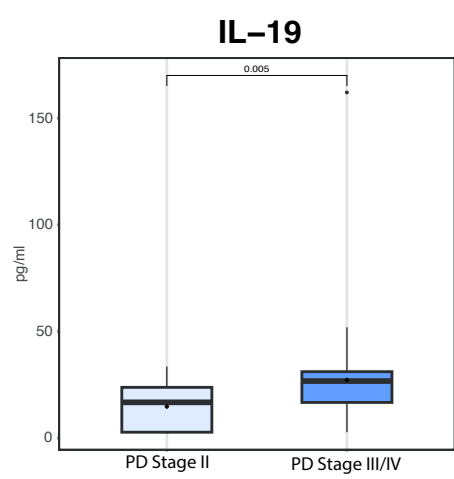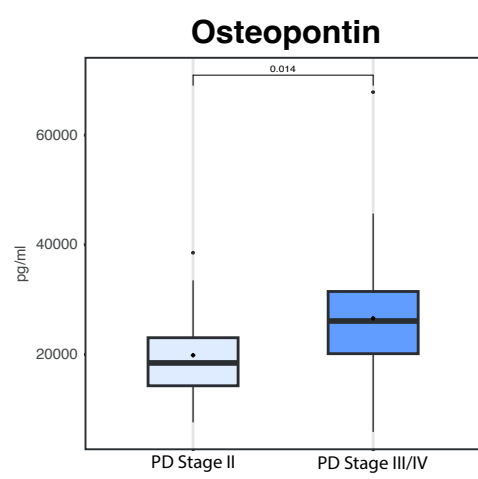

Supplement: Supporting Information 1 — Figure S1: Levels of inflammatory mediators in saliva (A) and serum samples (B), when comparing periodontitis stage II with stage III/IV. APRIL/TNFSF13, a proliferation-inducing ligand; BAFF/TNFSF13B, B-cell activating factor; IFN-α2, interferon alpha 2; IL, interleukin; PD, periodontitis; sCD30/TNFSF8, soluble CD30. [file 7739833.f1.pdf]
