# Supplementary material for: Comparative Analysis of Salivary and Serum Inflammatory Mediator Profiles in Patients With Rheumatoid Arthritis and Periodontitis
Source: Mediators Inflamm. 2025 Mar 20;2025:7739833. doi: 10.1155/mi/7739833 (PMC11949604; doi:10.1155/mi/7739833)
Supplement: Supporting Information 2 — Table S1: Medication of all participants, divided into two groups, ACPA-positive and ACPA-negative. [file 7739833.f2.docx]

**Supporting Information Table S1.** Medication of all participants (n=62), divided into two groups, ACPA-positive (n=48) and ACPA-negative (n=14).

|  | **ACPA-positive** | **ACPA-negative** |  |
| --- | --- | --- | --- |
| **Medication, no (%)** | **(n=48)** | **(n=14)** | ***p*** |
| Analgesics | 27 (56.3) | 5 (35.7) | 0.158 |
| NSAID | 15 (31.3) | 2 (14.3) | 0.217 |
| DMARDs | 32 (66.7) | 10 (71.4) | 0.742 |
| bDMARDs | 20 (41.7) | 4 (28.6) | 0.355 |
| Glucocorticoids | 21 (43.8) | 7 (50.0) | 0.731 |

Analyses were performed using independent t-test.
Abbreviations: ACPA, anti-citrullinated protein antibody; bDMARDs, biologic disease modifying anti-rheumatic drugs; DMARDs, disease modifying anti-rheumatic drugs; NSAID, non-steroidal anti-inflammatory drugs.
